# Viral infection collapses intracytoplasmic membrane integrity and autotrophic metabolism in ammonia-oxidizing *Nitrosomonas europaea*

**DOI:** 10.1093/ismeco/ycag170

**Published:** 2026-06-18

**Authors:** János Papendorf, David K Ngugi, Petra Büsing, Nicole Reimann, Johannes Wittmann, Stephanie Peter, Richard L Hahnke, Sarah Kirstein, Boyke Bunk, Manfred Rohde, Mathias Müsken, Meina Neumann-Schaal, Michael Pester

**Affiliations:** Leibniz Institute DSMZ-German Collection of Microorganisms and Cell Cultures, Inhoffenstraße 7B, Braunschweig 38124, Germany; GEOMAR Helmholtz Centre for Ocean Research Kiel, Wischofstraße 1-3, Kiel 24148, Germany; Leibniz Institute DSMZ-German Collection of Microorganisms and Cell Cultures, Inhoffenstraße 7B, Braunschweig 38124, Germany; Leibniz Institute DSMZ-German Collection of Microorganisms and Cell Cultures, Inhoffenstraße 7B, Braunschweig 38124, Germany; Leibniz Institute DSMZ-German Collection of Microorganisms and Cell Cultures, Inhoffenstraße 7B, Braunschweig 38124, Germany; Leibniz Institute DSMZ-German Collection of Microorganisms and Cell Cultures, Inhoffenstraße 7B, Braunschweig 38124, Germany; Leibniz Institute DSMZ-German Collection of Microorganisms and Cell Cultures, Inhoffenstraße 7B, Braunschweig 38124, Germany; Leibniz Institute DSMZ-German Collection of Microorganisms and Cell Cultures, Inhoffenstraße 7B, Braunschweig 38124, Germany; Leibniz Institute DSMZ-German Collection of Microorganisms and Cell Cultures, Inhoffenstraße 7B, Braunschweig 38124, Germany; Central Facility for Microscopy, Helmholtz Centre for Infection Research, Inhoffenstraße 7, Braunschweig 38124, Germany; Central Facility for Microscopy, Helmholtz Centre for Infection Research, Inhoffenstraße 7, Braunschweig 38124, Germany; Leibniz Institute DSMZ-German Collection of Microorganisms and Cell Cultures, Inhoffenstraße 7B, Braunschweig 38124, Germany; Braunschweig Integrated Centre of Systems Biology (BRICS), TU Braunschweig, Rebenring 56, Braunschweig 38106, Germany; Leibniz Institute DSMZ-German Collection of Microorganisms and Cell Cultures, Inhoffenstraße 7B, Braunschweig 38124, Germany; Chair of Microbial Physiology, Technical University of Munich, Emil-Ramann-Str. 4, Freising 85354, Germany

**Keywords:** nitrification, *Nitrosomonas*, phage, wastewater treatment plant, host response, transcriptome, metabolome

## Abstract

Ammonia-oxidizing bacteria (AOB) catalyze the first and rate-limiting step of nitrification. They are essential for nitrogen cycling in engineered and natural environments, yet little is known about their viruses or the consequences of phage infection for host physiology. Here, we report the isolation and characterization of a novel lytic bacteriophage, vB_NeuP-Nir1 (DSM 111086), infecting the model AOB *Nitrosomonas europaea*. Phage Nir1 ceased ammonia oxidation within hours, and caused complete lysis of host populations even at multiplicities of infection as low as 10^−6^. Electron microscopy revealed drastic host cell remodeling during infection, including pronounced cell bloating and large-scale disintegration of intracytoplasmic membranes. Integrated transcriptomic and metabolomic analyses showed that loss of these ATP and reducing equivalent generating membrane systems was accompanied by signatures of compromised lipid homeostasis and collapse of autotrophic CO₂ fixation. In parallel, Nir1 infection induced metabolic rewiring of the host, including upregulation of uptake systems for nucleic acids, amino acids, and small organic compounds, increased expression of iron acquisition and putative iron-dependent respiratory components, as well as accumulation of metabolites associated with membrane breakdown and stabilization of viral DNA. Together, these results provide the first detailed mechanistic insight into phage-induced host modulation in a chemolithoautotrophic nitrifier. Our study establishes the Nir1–*N. europaea* system as a model for investigating virus–host interactions in AOB and lays the foundation for assessing the role of phages in shaping nitrification and nitrogen cycling in engineered and natural ecosystems.

## Graphical Abstract

**Graphical Abstract:**
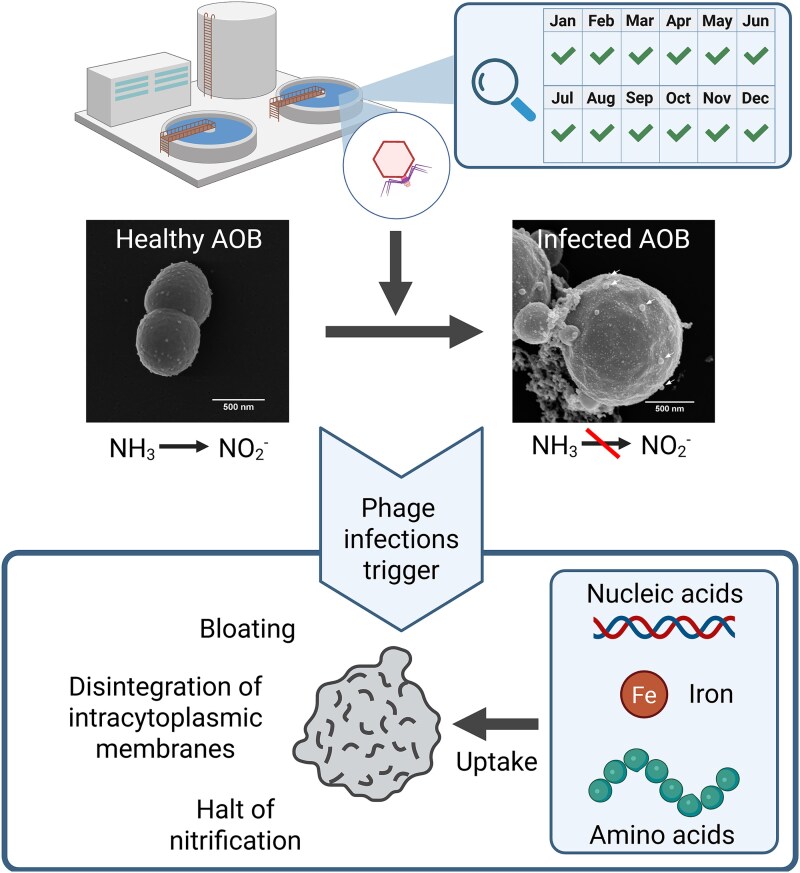

## Introduction

The widely distributed process of biological nitrification is an important part of the global nitrogen cycle. It describes the aerobic conversion of ammonia via nitrite to nitrate and occurs in marine, terrestrial, and engineered ecosystems [1]. In ammonia-rich environments, such as agricultural soils, the transformation of ammonia to leachable nitrate facilitates fertilizer loss and can ultimately lead to eutrophication of neighboring aquatic ecosystems [2]. Contrasting these undesirable effects, nitrification is an essential part of biological nitrogen removal from wastewater [3], which enables discharge of the effluent without causing eutrophication of affected water bodies and harm to aquatic life. Depending on niche adaptation, different ammonia-oxidizing microorganisms (AOMs) perform the first and rate-limiting step of nitrification [4–7]. Ammonia-oxidizing bacteria (AOB) are typically abundant in ammonia-rich environments [8, 9]. In contrast, different lineages of ammonia-oxidizing archaea (AOA) vary in their substrate concentration preference across a broad range of ammonia concentrations, including a strong predominance in low-ammonia environments [6]. Owing to their ability of complete ammonia oxidation to nitrate, a recently discovered third group of AOM was named Comammox [10, 11]. Similar to part of AOA, Comammox have been associated with an oligotrophic lifestyle [12] and found in low-ammonia environments [13, 14].

Representatives of all three AOM groups have been detected in wastewater treatment plants (WWTPs) [15–18]. Nevertheless, AOB often dominate the WWTP AOM-community at elevated ammonia concentrations with most representatives being affiliated with the genus *Nitrosomonas* [16, 19, 20]. *Nitrosomonas europaea* is common in WWTPs and serves as a model AOB [8]. Much of what is known about the physiology, biochemistry, and molecular biology of AOB is based on studies of this chemolithoautotrophic Betaproteobacterium [21]. It tolerates high concentrations of ammonia (up to 100 mM) and nitrite (up to 25 mM) [21]. Due to the toxicity of intermediates and products (i.e. hydroxylamine, nitric oxide, and nitrite) of its energy metabolism, the respective enzymatic machinery is located mainly in the membrane, coupled with the release of metabolites into the periplasmic space [22]. This explains why AOB including *N. europaea* are characterized by a high degree of intracytoplasmic membrane stacks to increase the surface for their energy metabolism [23]. *N. europaea* typically grows autotrophically by utilizing the Calvin cycle, although there are reports of *N. europaea* growing as a facultative chemolithoheterotroph with fructose or pyruvate as a resource for its anabolic pathways [24]. Altogether, this knowledge makes *N. europaea* a highly suitable model organism to study principles of virus–host interactions for AOM in high-ammonia settings.

Viruses that infect prokaryotes are omnipresent and abundant in the environment [25–27] and man-made systems like WWTPs [28]. It has been postulated and partially demonstrated that they play an important role in shaping microbial communities and associated carbon cycling [29–32]. However, little is known about the impact of viruses on nitrogen cycling and especially AOM. Regardless of clade, the growth of AOM including *N. europaea* is slow and generally only occurs in liquid cultures or takes multiple weeks on solidified medium [33, 34]. This renders typical plaque-based virus isolation methods ineffective and necessitates alternative techniques. It also explains why virus–host interactions have been studied very little in these chemolithoautotrophic microorganisms and mainly relate to descriptions in metagenomic datasets [35–37]. So far, only two AOM-infecting viruses—a chronically AOA-infecting virus [38] and a lytic AOB-infecting bacteriophage [39]—have been isolated. However, studies targeting viral host modulation of AOM are completely lacking. In this study, we isolated a bacteriophage infecting *N. europaea* from a WWTP and provide the first integrated analysis of phage-induced host modulation in a chemolithoautotrophic nitrifier.

## Material and methods

### Isolation and genome analysis of phage vB_NeuP-Nir1

*N. europaea* strain Nm50^T^ (=DSM 28437) represents the neotype strain of *N. europaea* Winogradsky 1892 (Approved Lists 1980) [40, 41]. It was grown for 19 days in 100 ml of DSMZ medium 1583 (Supplementary Material) at 28°C in the dark without agitation. Thereafter, the culture was concentrated by centrifugation for 15 min at 14 334 × *g* and 15°C, followed by resuspension in 1 ml of the supernatant. Initial infection of the bacterial culture was performed by adding 8 ml of fresh DSMZ medium 1583 and 1 ml of pre-filtered (0.22 μm pore size, polyethersulfone, Corning®, Corning Inc., Corning, NY, USA) activated sludge obtained on 2 December 2019 from the WWTP Steinhof located in Braunschweig, Lower Saxony, Germany (52°19′05.9″N, 10°26′45.1″E). Positive infection was indicated by the absence of ammonia oxidation activity, which otherwise results in a pH drop of the medium and can be monitored by the included pH indicator cresol red. Isolation of the bacteriophage was performed by repeated serial dilution of 1 ml of infected host cultures in 10 ml culture tubes containing 8 ml of fresh DSMZ medium 1583 and 1 ml of the host strain grown for about 2 weeks in DSMZ medium 1583 before infection.

Purity of the isolated phage was confirmed by electron microscopy and sequencing of the phage genome as detailed in Supplementary Material. For the latter, short-read Illumina (mean coverage 647×) and long-read Oxford Nanopore Technologies (ONT) (mean coverage 23×) sequencing were combined. Assembly of SUP (super accurate) base-called reads from ONT sequencing was done using Flye [42, 43] version 2.9. In Geneious Prime version 2025.0.2, reads produced via Illumina sequencing were Q30-trimmed and filtered for a minimal length of 10 nucleotides using BBDuk (part of BBMap suite) v38.84 [44]. Short reads were then mapped to the assembled contig using Bowtie 2 version 2.4.5 [45] to correct errors from ONT sequencing. Termini of the phage genome could not be fully resolved. Only sequence information was considered for the final viral contig where both ONT and Illumina data were available. Gene annotation was done with Pharokka version 1.7.5 [46]. More sensitive annotation of hypothetical genes was done with Phold version 0.2.0 [47]. The start site of one coding DNA sequence (CDS) was manually corrected to begin with a start codon. A circular plot of the viral genome was created using the plot option in Phold [47]. Phylogenomic reconstruction was done in ViPTree (Viral Proteomic Tree) server version 4.0 [48]. BIONJ-based [49] tree calculation was done using default settings but with additional gene functional prediction using GHOSTX. Calculation of intergenomic similarities between phages was performed using the web servers of taxMyPhage v3.3.6 [50] and of VIRIDIC [51] with default parameter settings. Host range experiments and a diagnostic polymerase chain reaction (PCR) assay for the gene encoding the major head protein of Nir1 are detailed in Supplementary Material.

### Defined infection experiments

Before onset of infection experiments, phage concentrations were determined by counting SYBR Gold (Invitrogen, Life Technologies Corporation, Eugene, OR, USA) stained phages on a 0.02 μm pore-size Anodisc aluminum oxide filter membrane (Whatman™, Global Life Sciences Solutions Operations UK Ltd, Little Chalfont, UK) using 100 randomly selected fields of 20 × 20 μm or 400 fields of 10 × 10 μm. Phage staining was performed as described in Kim *et al*. [38] with minor modifications (Supplementary Material). We monitored bacterial growth and nitrite production from 43 h before to 29–121 h after phage addition at different multiplicities of infections (MOIs). Host populations were followed by flow cytometry. Sample preparation of bacterial populations consisted of filtration through a 10 μm cell-sieve (CellTrics™, Sysmex Partec GmbH, Görlitz, Germany) to remove medium precipitates, followed by bacterial staining for 10 min in the dark using 1 μl of 500 μM SYTO-9 (Invitrogen) added to 999 μl of bacterial culture. Bacterial concentrations were determined using a CytoFLEX S benchtop flow cytometer (Beckman Coulter, Brea, CA, USA) and co-analysis with 4 μm CountBright Plus Absolute Counting Beads (Invitrogen) as an internal standard. For each sample, 50 000–100 000 events were recorded using a threshold of 4000 in the SSC-H channel and adjusted gain settings (forward scatter FSC = 120, side scatter SSC = 700, SYTO-9 = 1750). Events representing bacterial cells and counting beads were captured in gates created in SYTO-9 vs. SSC and FSC vs. SYTO-9 plots, respectively. Nitrite concentrations were quantified using a S150 Ion Chromatography System (SYKAM Chromatographie Vertriebs GmbH, Fürstenfeldbruck, Germany), equipped with a SykroGel AX300 column, using 0.025 mM NaSCN and 4 mM Na_2_CO_3_ as eluent at a flow rate of 1 ml min^−1^.

### Transcriptome response of Nm50^T^ to Nir1-infection

Isolation of genomic DNA and de-novo sequencing of a closed reference genome of the host Nm50^T^ were performed as described in Supplementary Material. Bacterial and viral concentrations were quantified as described above. A 2.5 L culture of strain Nm50^T^ was grown statically in a 5 L Erlenmeyer flask for 11 days at 28°C in the dark. The bacterial culture was split into six 1 L Erlenmeyer flasks, each containing 290 ml of the culture. Three of the replicates were amended with 7.38 ml of the phage lysate of Nir1 (MOI = 4.1) and gently mixed, the remaining three replicates served as controls. Infected and non-infected cultures were sampled directly after phage addition and after 120 min of static incubation at 28°C in the dark. For each timepoint and replicate, 140 ml of culture were collected on ice and directly centrifuged in 250 ml polypropylene copolymer (PPCO) centrifuge bottles (Nalge Nunc International Corporation, Rochester, NY, USA) for 30 min at 4°C and 9000 × *g*. The supernatant was carefully decanted, and the pellet was directly resuspended in 600 μl lysis buffer of the AllPrep PowerViral DNA/RNA Kit (Qiagen, Hilden, Germany).

RNA extraction was performed immediately after sampling as described by the manufacturer. Extracted nucleic acids were eluted in molecular grade, nuclease-free water (Ambion™, Life Technologies Corporation, Austin, TX, USA), and immediately frozen at −80°C until further analysis. The presence of RNA and DNA was confirmed via agarose gel electrophoresis using 1% w/v Wide Range agarose (SERVA Electrophoresis GmbH, Heidelberg, Germany) in 1× Tris-acetate-EDTA (TAE) buffer (Carl Roth GmbH + Co. KG, Karlsruhe, Germany). DNA was then removed using recombinant DNase I treatment (DNA-*free*™, Invitrogen) following the manufacturer’s instructions. Purified RNA was quantified using the Qubit™ RNA HS Assay Kit (Invitrogen) according to the manufacturer’s instructions. Removal of genomic DNA was confirmed by 16S rRNA gene PCR (35 cycles) using the general bacterial primers 27-f (5′-AGAGTTTGATYMTGGCTC-3′) and 1492-r (5′- GGYTACCTTGTTACGACTT-3′) at an annealing temperature of 52°C. Absence of specific PCR products was taken as a sign of efficient DNA removal.

For transcriptome sequencing, 50 μl of purified RNA were first concentrated using the RNA Clean & Concentrator™-5 kit (Zymo Research, Irvine, CA, USA), then depleted for rRNA using the Ribo-off rRNA Depletion Kit for Bacteria (Vazyme Biotech, Nanjing, PRC), and finally used to prepare Illumina sequencing libraries with the TruSeq Stranded mRNA kit (Illumina, San Diego, CA, USA). Quality of libraries was confirmed via nucleic acid fragment analysis using a Femto Pulse System (Agilent Technologies, Inc., Santa Clara, CA, USA). Sequencing was performed on a NextSeq 2000 Sequencing System (Illumina) with a 2 × 150 bp paired end read length. To remove sequences of insufficient quality, reads were Q30-trimmed and filtered to a minimum length of 100 nucleotides using BBDuk (implemented in BBMap toolkit suite v37.62) [44], followed by discarding singletons. Reads were then mapped to the host and phage CDS using BBMap v37.62 [44] with the settings minid = 0.99 and local = t. Absolute read counts were extracted from the resulting mapping statistics files and used for differential expression analysis of host genes using the DESeq2 package v1.46.0 [52] with standard settings for a one-factorial design in R v4.4.1 (https://cran.r-project.org/). Visualization of differentially expressed genes was performed using the EnhancedVolcano package v1.24.0 (https://github.com/kevinblighe/EnhancedVolcano) in R v4.4.1.

### Metabolome response of Nm50^T^ to Nir1-infection

Bacterial and viral concentrations were quantified as described above. Two 1.8 L Nm50^T^ cultures were grown statically in 5 L Erlenmeyer flasks for 12 days at 28°C in the dark. Directly prior to the experiment, they were combined and 290 ml aliquots were distributed to 12 1 L Erlenmeyer flasks. Six replicates served as uninfected controls, and six replicates were amended with 11.4 ml phage lysate of Nir1 (MOI = 3.44) for the infection experiment. Sampling was performed directly at the start of each treatment and after 120 min of static incubation at 28°C in the dark. Since sampling from six replicates per treatment requires a short time period that includes first phage activity, we defined the timepoint at the start of the infection experiment as early response and the timepoint after 120 min as late response. For each timepoint and replicate, 140 ml of culture were centrifuged in 250 ml PPCO centrifuge bottles (Nalge Nunc International Corporation) for 30 min at 4°C and 9000 × *g*. The supernatant was carefully decanted, the pellet resuspended in 1 ml of fresh DSMZ medium 1583, and centrifuged again for 15 min at 4°C and 9000 × *g*. The supernatant was removed with a pipette, the biomass immediately frozen in liquid nitrogen, and stored at −80°C.

For gas chromatography-mass spectrometry (GC–MS) analysis, the biomass of each replicate was extracted by adding 250 μl of methanol, spiked with 0.5% ribitol (0.2 mg ml^−1^), for 15 min at 70°C in an ultrasonic bath. Samples were incubated for 2 min on ice before 250 μl of water were added. Samples were mixed vigorously. Subsequently, 300 μl dichloromethane were added and the samples were mixed again. Following a centrifugation step (5 min, 10 000 × *g*), the polar phase was collected and 400 μl were dried under vacuum. Samples were derivatized and measured on a gas chromatograph-mass selective detector (GC–MSD) system (7890B coupled to a 5977 GC, Agilent Technologies, Inc.) equipped with a high-efficiency source and an RTC system (GERSTEL GmbH & Co.KG, Mülheim an der Ruhr, Germany) as described before [53]. In brief, a two-step derivatization with a methoxyamine hydrochloride solution (20 mg ml^−1^ in pyridine) and *N*-methyl-*N*-(trimethylsilyl)-trifluoroacetamide was performed automatically, followed by separation on a VF-5 ms column (Agilent Technologies, Inc.) and analysis in the scan mode. Data analysis of intracellular metabolites was performed as previously described [54, 55]. Metabolites were manually assigned to Kyoto Encyclopedia of Genes and Genomes (KEGG) metabolic pathways [56]. Graphs and Kruskal Wallis nonparametric tests with Dunn’s multiple comparisons tests were performed using the Graph function of BioRender (https://biorender.com).

## Results

### A novel podovirus within the *Autographivirales*

**Figure 1:**
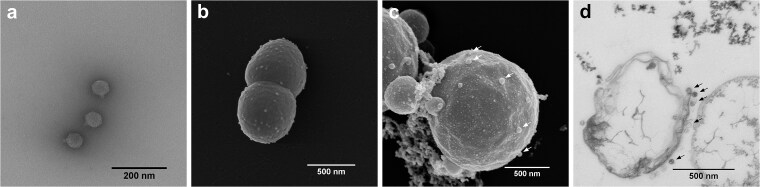
Morphology of bacteriophage vB_NeuP-Nir1 (Nir1) as well as uninfected and Nir1-infected cells of *N. europaea* Nm50^T^; (a) TEM of negatively-stained, podovirus-shaped bacteriophage Nir1, (b) SEM of uninfected *N. europaea* Nm50^T^ cells showing typical morphology, (c) SEM of Nir1-infected *N. europaea* Nm50^T^ cells showing a bloated morphology and vesicle production, white arrows mark representative virions (d) ultrathin sections of Nir1-infected *N. europaea* Nm50^T^ cells, recorded via TEM, showing disintegrated intracellular membranes and missing cytoplasmic content, black arrows mark representative virions.

**Figure 2:**
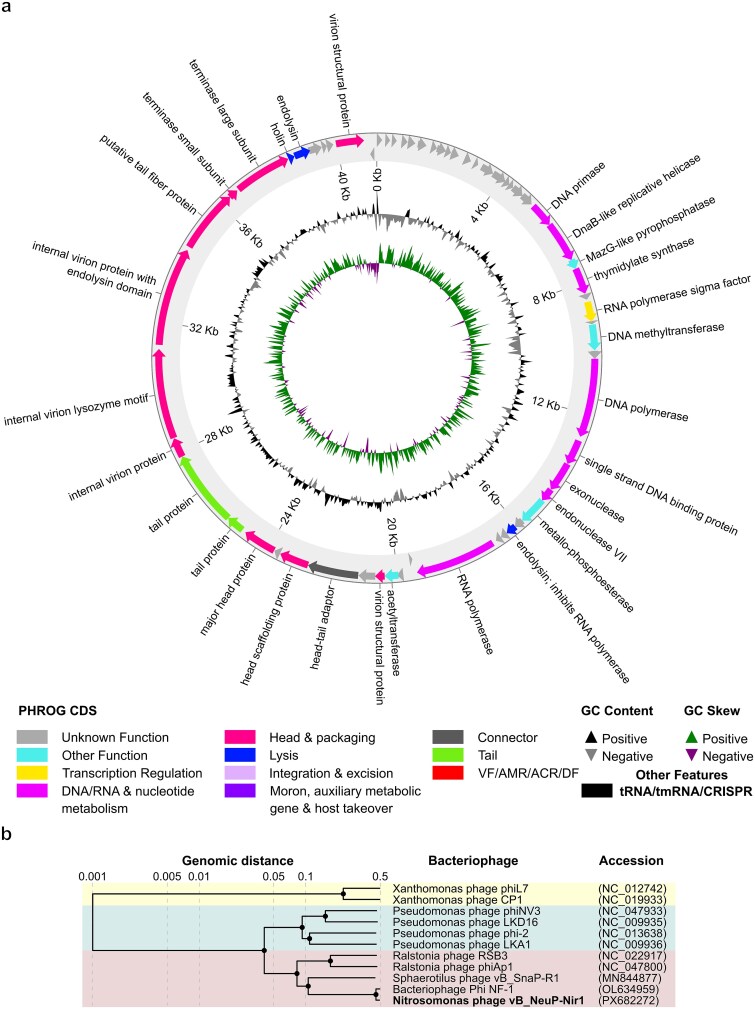
Genome characteristics and proteomic similarity analysis of bacteriophage vB_NeuP-Nir1; (a) genome organization and annotated genes of bacteriophage vB_NeuP-Nir1, (b) BIONJ-based [49] user-generated viral proteomic tree created from genomic distances derived from normalized tBLASTx scores [48]; genomic nucleotide sequences of vB_SnaP-R1, ΦNF-1, and vB_NeuP-Nir1 were uploaded to the ViPTree server using dsDNA as nucleic acid type and prokaryote as host category of reference viruses; representative closely related phages of the family *Autoscriptoviridae* and an outgroup (*Xanthomonas* phages phiL7 and CP1) of the class *Caudoviricetes* were manually selected from the complete sequence list provided with the calculated tree and used to create a user-generated tree.

We isolated the lytic bacteriophage vB_NeuP-Nir1 (hereafter Nir1) infecting the AOB *N. europaea* Nm50^T^ (hereafter Nm50^T^) by amending an actively growing host culture with 0.2-μm-filtered wastewater followed by serial dilution. Electron microscopy revealed a podovirus shape of virions comprising a 61.5 ± 2.9 nm × 61.5 ± 2.8 nm (length × diameter, mean ± SD, *n* = 13) capsid and a 13.2 ± 1.1 nm (length, mean ± SD, *n* = 13) tail (Fig. 1a). We sequenced its 41 212 bp-long genome and were able to annotate 29 of the 61 predicted protein-coding genes (Fig. 2a). A ViPTree-based [48] comparison of genome-wide similarities computed by tBLASTx revealed the closest relatedness to bacteriophages of the order *Autographivirales* [57], which infect *Pseudomonadota* members (Fig. 2b). According to the Virus Intergenomic Distance Calculator VIRIDIC [51], Nir1 shares 90.6% intergenomic similarity with ΦNF-1, the first described phage infecting multiple *Nitrosomonas* species [39], but only 4.6% similarity with the next closest related phage vB_SnaP-R1 (=SnaR1), infecting heterotrophic *Sphaerotilus natans* strain DSM 6575 [58]. With 90.8% calculated similarity between Nir1 and ΦNF-1, results from the taxonomic assignment tool taxMyPhage [50] were highly consistent. Following the demarcation thresholds of the International Committee on Taxonomy of Viruses (ICTV) [59], both, taxMyPhage and VIRIDIC identified Nir1 as a novel species in the recently established genus *Catalonvirus*, which currently contains solely species ΦNF-1. We further followed the presence of the gene encoding the major head protein of Nir1 in the WWTP Steinhof throughout the year using a diagnostic PCR. Specific PCR products were detectable year-round but were most pronounced from July to October (Supplementary Fig. S1). Nir1 was deposited at DSMZ under the accession number DSM 111086.

### Nir1 severely alters host morphology

Upon infection with Nir1, cells of Nm50^T^ undergo a drastic morphological change from short coccoid rods (Fig. 1b), with a typical cell volume of 0.17 ± 0.05 μm^3^ (mean ± SD, *n* = 17), toward a swollen spherical phenotype (sporadically with vesicular membrane extrusions, Fig. 1c), with a 4.5-fold larger volume of 0.76 ± 0.34 μm^3^ (mean ± SD, *n* = 16). Occasionally, aggregation of cells occurred in infected cultures. To investigate intracellular changes in infected bloated host cells, we performed transmission electron microscopy on cell section. We observed that intracytoplasmic membranes as characteristic for *Nitrosomonas* species [60] and other nitrifiers [23] were disintegrated after infection was completed. Virions were not detected within infected bloated host cells (Fig. 1d), unless infection deemed incomplete, indicating that progeny is generally efficiently released.

**Figure 3:**
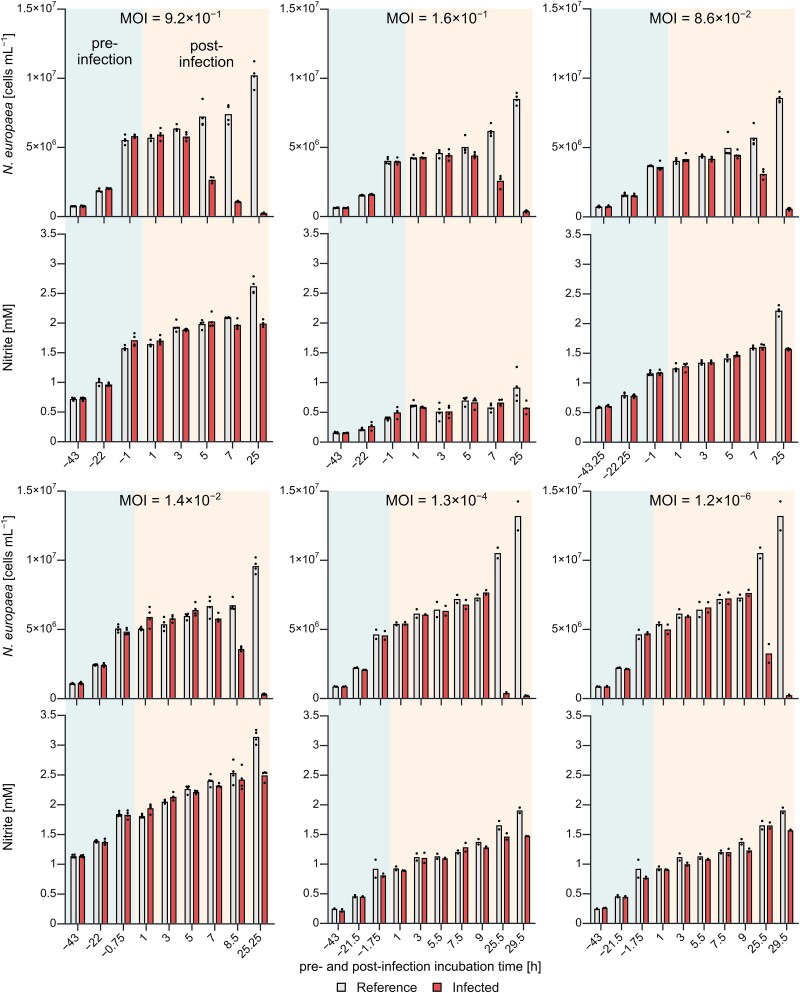
Infection experiments of *N. europaea* Nm50^T^ with phage vB_NeuP-Nir1 under different multiplicities of infection (MOI). Host cell numbers and nitrite concentration are shown as proxies for bacterial growth and activity, respectively (*n* = 4; except for MOIs 1.3 × 10^−4^ and 1.2 × 10^−6^, *n* = 2).

To test the ability of Nir1 to infect alternative hosts, we screened four additional AOB species of the genera *Nitrosomonas* (average nucleotide identities of 69.67%–79.26% to Nm50^T^) and *Nitrosospira* (average nucleotide identity of 68.83% to Nm50^T^), including all described host species of ΦNF-1 [39]. Only a minor fraction of *Nitrosomonas nitrosa* DSM 28438 (=Nm90^T^) cells showed typical signs of infections (i.e. hollow or bloated cells), while all other screened hosts were negative for infection. To analyze the virulence of Nir1, infection experiments with defined virus-to-host ratios were performed in liquid cultures of Nm50^T^ with MOIs ranging from 9.2 × 10^−1^ down to 1.2 × 10^−6^. Here, MOI is defined as the ratio of phage particles to host cells at the time of inoculation. At the highest MOI tested, lysis began ~3 h post infection, which coincided with a cessation of nitrite production, the proxy for the metabolic activity of AOB. Lowering the MOI gradually slowed the lysis of the host population, mirrored by delayed halt of nitrification. However, even at the lowest MOI tested (1.2 × 10^−6^), metabolic activity ceased after 25.5 h and complete lysis of the host population was observed 29 h post infection (Fig. 3).

### Nir1 infection triggers overexpression of genes encoding membrane-associated proteins

We next analyzed host gene expression during the early (right after infection) and late (120 min after infection) latent periods of Nir1. Representative time points were chosen as based on a detailed analysis of the latter until lysis set in (Supplementary Fig. S2.) This was conducted at an MOI of 4.1 to ensure a high proportion of synchronously infected host cells. In addition, de-novo sequencing of Nm50^T^ was done to ensure a closed circular genome and that responses pertaining to the isotype deposited at DSMZ and used in our experiments are not missed, while being aware that another *N. europaea* strain, ATCC 19718, has been already genome sequenced and carefully annotated before [61]. Genes were considered significantly differentially expressed (adjusted *P*-value <0.05) if log_2_-fold changes (LFC) were above +1 or below −1.

**Figure 4:**
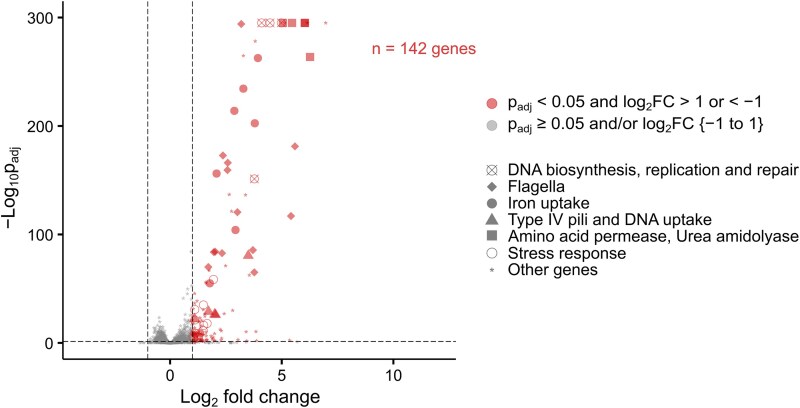
Differentially expressed genes of Nir1-infected *N. europaea* Nm50^T^ in comparison to uninfected controls; samples were taken at a timepoint corresponding to the late infection process (120 min post infection, *n* = 3); log_2_-fold change (LFC) cut-offs were set to +1 and −1 for up- and downregulated genes, respectively, and are represented by dashed vertical lines; adjusted *P*-values (*P*_adj_) are shown as −Log_10_*P*_adj_; the significance cut-off for adjusted *P*-values of 0.05 is represented by a dashed horizontal line; significantly differentially expressed genes are marked in red, all other genes are shown in gray.

At the early infection timepoint, comparison of Nir1-infected to non-infected cells did not reveal significant differential expression of genes. At the late infection timepoint, Nir1-infected cells showed significant transcriptional upregulation of 142 genes compared to non-infected cells but no downregulation (Fig. 4, Supplementary Table S1). Two genes coding for multicopper oxidases showed high overexpression (LFC of 6.16 and 6.14, respectively), with four additional genes coding for redox-active proteins, including cytochrome c, being overexpressed as well (LFC 1.19–1.61). Further transcriptionally highly upregulated genes (LFCs 5.05–6.27) encoded an amino acid permease, a urea carboxylase, and three urea amidolyase gene products. With LFCs ranging between 1.35 and 3.93, we detected the overexpression of eight genes involved in iron uptake or associated with low iron availability.

Notably, two genes coding for flagellar transcriptional activators (*flhD* and *flhC*) with an adjacent gene coding for a ComEC/Rec2 protein (LFC 3.50) and a gene cluster consisting of at least 12 flagellar genes (*flgABCDEFGHIJKL*) were highly upregulated as well (LFCs 1.01–5.59). Furthermore, we detected four overexpressed genes (LFCs 1.10–2.04) involved in Type IV pilus biogenesis (*pilP, pilO, pilN*, and *pilQ*). We also observed the overexpression of one gene coding for carbon starvation protein A (LFC 2.65) and, directly adjacent, one gene coding for an YbdD/YjiX family protein (LFC 2.80). Other categories of overexpressed genes include those involved in stress response, DNA synthesis, replication and repair, transcriptional regulation, ribosomal proteins, and cell wall remodeling (Supplementary Table S1). In addition, 27 upregulated genes coded for proteins of unknown function and 11 for transposases. Investigation of infected cells via electron microscopy did not reveal enhanced production of cellular appendices such as flagella or pili but confirmed onset of bloating and vesicle formation 3 h post infection.

### Nir1 infection increases membrane-associated metabolite levels

**Figure 5:**
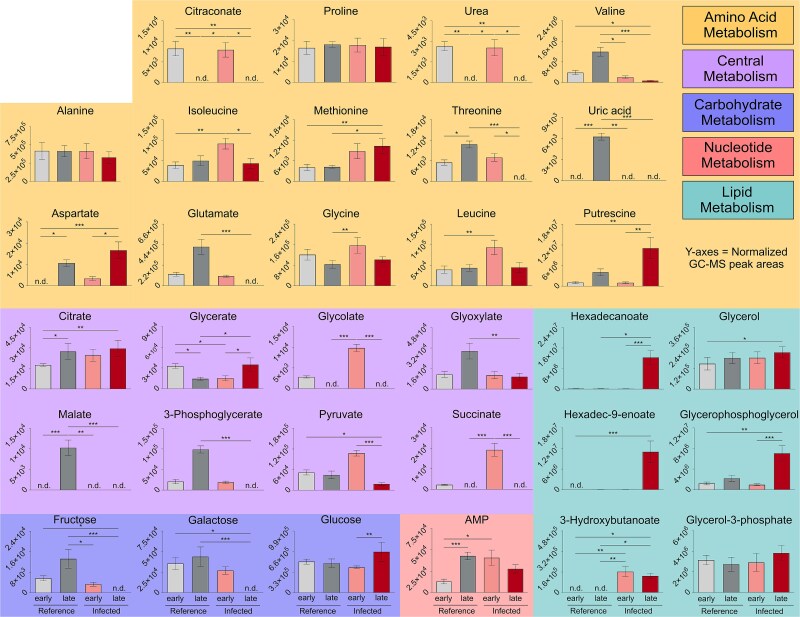
Metabolite profiles of Nir1-infected and uninfected *N. europaea* Nm50^T^ cultures; samples were taken during the early (shortly after infection) and late (120 min) infection process as well as corresponding time points for uninfected cultures; uninfected cultures are shown in light or dark gray, Nir1-infected cultures are shown in light or dark red, representing early or late infection time points, respectively; values represent normalized peak areas of metabolites detected using GC–MS (*n* = 6, except for early infected cultures, *n* = 5), error bars represent standard deviation, n.d. = not detected; pairwise comparisons were performed using Dunn’s multiple comparisons test with Bonferroni correction of *P*-values following Kruskal-Wallis tests; assigned significance annotations represent the following thresholds: ^*^ = *P*_adj_ ≤ .05, ^**^ = *P*_adj_ ≤ .01, ^***^ = *P*_adj_ ≤ .001.

To elucidate how infections of Nir1 impact the central metabolism of Nm50^T^, we analyzed changes in its respective metabolite profile during the early and late infection process using the same experimental setup as done for the transcriptomic responses. In total, profiles of 32 metabolites could be detected (Fig. 5) and assigned to KEGG metabolic pathways. Concentrations of metabolites associated with lipid- and fatty acid metabolism were strongly and significantly elevated in Nir1-infected cultures compared to their controls. This effect was specifically pronounced for glycerophosphoglycerol and 3-hydroxybutanoate, respectively. We also detected strong and significant increases of the fatty acids hexadecanoate and hexadec-9-enoate, further supporting changes in membrane-associated metabolites. A second group of significantly altered metabolite profiles comprised the core central metabolism and encompassed pyruvate, 3-phosphoglycerate, malate, succinate, glyoxylate, and glycolate. While there was no common trend of up- or downregulation, the observed changes indicate altered routes of carbon flow within the Calvin cycle and the biosynthesis-supporting citric acid cycle and gluconeogenesis of these autotrophic bacteria. The third major group of altered metabolite profiles included various amino acids, as would be expected from a shifted amino acid demand of proteins involved in the infection process, including phage-specific proteins. However, altered amino acid profiles might also be linked to an alteration of the central metabolism in infected cells. In addition, the polyamine putrescine had significantly higher cellular concentrations in infected cells at the late infection process timepoint. Other metabolites with altered concentration profiles included the monosaccharides glucose, fructose, and galactose as well as AMP as a metabolite directly involved in energy metabolism.

## Discussion

Phages have been shown to modulate the transcriptome and metabolome of heterotrophic and phototrophic hosts to varying extents [62–65]. We extend this knowledge to a chemolithoautotrophic microorganism involved in nitrification, a fundamental and nearly ubiquitous biogeochemical process in the N cycle [1]. We isolated Nir1 as a phage that causes complete lysis of ammonia-oxidizing *N. europaea* Nm50^T^. Nm50^T^ represents the neotype strain of *N. europaea* as the best-studied model species of the genus *Nitrosomonas* [21], with *Nitrosomonas* representatives being known for their presence and relevance in WWTPs [16, 19, 66]. The gene encoding the major head protein of Nir1 was detectable year-round in the WWTP Steinhof, with higher prevalence during summer months (Supplementary Fig. S1). This indicates that Nir1 or closely related phages are a stable part of the virome of this WWTP.

The recent isolation of the related *Nitrosomonas*-infecting phage ΦNF-1 from a Spanish WWTP [39] shows that AOB-infecting phages are likely an integral part of WWTPs. Both phages represent separate and, so far, the only known species of the genus *Catalonvirus*. However, despite having >90% identical genomes, the two phages exhibit different host ranges, with Nir1 efficiently lysing only *N. europaea* Nm50^T^, while ΦNF-1 is able to infect a broader AOB host range [39]. Electron microscopy revealed that Nir1-infected cells became bloated with a 4.5-fold increase in volume and frequently produced membrane vesicles, while characteristic intracellular membranes disintegrated (Fig. 1). This was paralleled by significantly increased concentrations of the C16-fatty acids hexadecanoate (palmitate) and hexadec-9-enoate (palmitoleate) (Fig. 5). In AOB, intracytoplasmic membranes result from invagination of the cytoplasmic membrane [23] and constitute a major structural and functional compartment, as they harbor the ammonia monooxygenase complex [67] and are highly enriched in C16 fatty acids (>96%), particularly palmitate and palmitoleate [68]. Increased levels of free C16-fatty acids are best explained by large-scale phospholipid hydrolysis resulting from viral takeover. Similar increases in dominant membrane fatty acids have been reported during T4-phage infection in *Escherichia coli*, reflecting the breakdown of host membranes and the release of lipid constituents [69, 70]. The concurrent accumulation of glycerophosphoglycerol (Fig. 5) as a major molecular backbone of phospholipids [71, 72], further supports intracellular membrane degradation by Nir1. We cannot exclude that the observed increases of fatty acids and glycerophosphoglycerol relate at least in parts also to the three-step lytic release of virions in Gram-negative bacteria [73, 74]. Specifically, spanins catalyze the breakdown of the outer membrane and thus the last step in virion release during this three-step lytic process. If spanins are absent, prior endolysin-mediated degradation of the cell wall (step 2 of lytic release) can result in spherical cells [75, 76] as seen for Nm50^T^. However, in this case, we should have observed pronounced trapping of viral progeny in bloated Nm50^T^ cells after infection, which was not the case (Fig. 1d).

Accumulation of fatty acids in their free form can disrupt membrane integrity, impair the respiratory electron transport chain, and inhibit membrane-bound enzymes [77]. In addition to the large-scale loss of intracytoplasmic membranes, such effects would further compromise energy conservation in Nm50^T^, given the tight coupling between ammonia oxidation, proton motive force generation, and membrane integrity. Besides ATP, also reducing power required for CO_2_ fixation by the Calvin cycle is generated by membrane-bound electron transport linked to ammonia oxidation, rendering carbon fixation highly sensitive to loss of membrane integrity [67, 78]. Consistent with this, Nir1 infection was accompanied by significant decreases in 3-phosphoglycerate and glyoxylate (Fig. 5). In particular, 3-phosphoglycerate represents the primary stable product of RuBisCO-mediated CO₂ fixation [79], and its depletion likely reflects collapse of Calvin cycle throughput under ATP- and NAD(P)H-limiting conditions. Glyoxylate is directly connected to the oxygenase side activity of RuBisCO. It is a typical intermediate in the phosphoglycolate salvage pathway [80], and its depletion further supports collapse of RuBisCO activity. The concurrent decline in hexose intermediates, such as fructose and galactose, aligns with these observations as it suggests failure of upstream anabolic carbon flow.

Parallel accumulation of glucose may result from metabolic disruption of the interconnected Calvin cycle, gluconeogenesis, and oxidative and reductive pentose phosphate pathways, which share multiple enzymes and form a superpathway in *N. europaea* [81]. Such imbalance in the metabolic network may also explain increased levels of pyruvate and succinate (central carbon metabolism) as well as glycolate (phosphoglycolate salvage pathway) during the early infection process (Fig. 5). The reason for 3-hydroxybutanoate (3-hydroxybutyrate) accumulation is not clear. While 3-hydroxybutanoate is the well-established monomer of the storage compound polyhydroxybutyrate (PHB) [82], PHB accumulation in *Nitrosomonas* species has not been reported. PHB may have formed transiently and been used as carbon source by the host during Calvin cycle breakdown. Alternatively, 3-hydroxybutanoate may have accumulated due to metabolic disruption by Nir1, caused by incomplete processing of acetyl-CoA and reducing equivalents, which are then channeled toward 3-hydroxybutanoate as a sink for excess carbon and reducing equivalents [82].

Viral infection also altered the profiles of free amino acids, although changes were not very pronounced (Fig. 5). This is likely explained by the similar amino acid composition of the overall host proteome as compared to the overall phage proteome or even the amino acid composition of its major capsid protein (Supplementary Table S2). Intriguingly, the arginine-derived diamine putrescine was highly upregulated during the late infection process (Fig. 5). Along with spermidine and spermine, putrescine is one of the major polyamines in bacteria. These cationic compounds are known to bind DNA and benefit phage replication and genome packaging [65, 83, 84]. However, putrescine was also shown to support anti-phage responses in *Pseudomonas aeruginosa*, where it served as danger signal released by lysed cells and sensed by adjacent host cells [85]. Also, polyamines have been shown to be connected to the production of siderophores [86, 87] with iron limitation of Nir1-infected Nm50^T^ being indicated by our transcriptome analyses (Fig. 4).

Host transcriptome profiling identified the overexpression of genes involved in transcriptional regulation (RNA polymerase sigma factors) and translation (ribosomal proteins) as well as general stress responses, and DNA synthesis, replication, and repair (Fig. 4, Supplementary Table S1). Such responses are also known from the infection process of other lytic phages [88, 89]. In addition, genes involved in starvation response or nutrient uptake were overexpressed in Nir1-infected Nm50^T^ during the late infection process. This effect was pronounced for genes associated with iron scavenging. *N. europaea* is known to have a high iron demand, mainly for heme and Fe-S cluster containing enzymes of its respiratory chain [61, 90]. Since Nir1-infection caused a massive breakdown of intracytoplasmic membranes, scavenging for iron may have supported synthesis of iron-containing respiratory chain proteins to counterbalance production loss of ATP and reducing equivalents. Supporting this observation is the overexpression of genes encoding proteins involved in electron transfer, such as cytochrome c and multicopper oxidases (Supplementary Table S1). The latter have been proposed to be part of respiratory processes in AOA, possibly substituting for iron-containing cytochromes [91]. A starvation response is further indicated by the overexpression of one *cstA-* and one *ybdD-*related upregulated gene in late-infected cells (Supplementary Table S1). Both have been shown to play roles in the uptake of pyruvate in carbon-starved *E. coli* cells [92]. Previous studies revealed that pyruvate [93] and amino acids [94, 95] can support the growth of *N. europaea* cultures and that chemolithoorganotrophic growth on organic compounds, including pyruvate [24], is possible.

We observed that infections with Nir1 resulted in the overexpression of flagellar and type IV pilus genes in the late infection process. Interestingly, genes encoding the actual filament subunits (*flaA* and *pilA*) were only slightly overexpressed (LFC 0.33 and 0.57, respectively) in comparison to other genes of the flagella and pilus apparatus. This is supported by our electron microscopy investigation, which did not reveal a massive switch to a flagellated phenotype in Nm50^T^ cells. Type IV pili and flagella can serve multiple roles and are best known for facilitating motility. However, the hollow structure of type IV pili can also facilitate the uptake of extracellular DNA, which involves the presence of the highly conserved ComEC protein to enable transport through the cytoplasmic membrane [96]. We found a highly overexpressed gene coding for a ComEC/Rec2 type protein in the late infection process, indicating that DNA-uptake might play a role in Nir1-infected Nm50^T^ cells.

The parallel uptake of external amino acids was indicated by the overexpression of genes encoding an amino acid permease (Supplementary Table S1). Furthermore, we observed a strong overexpression of genes coding for a urea carboxylase and urea amidolyase-related proteins (Fig. 4, Supplementary Table S1). Interestingly, Nm50^T^ lacks genes encoding the classical urease [61]. Urea carboxylase is part of the urea amidolyase complex and can be used as an alternative to urease for the conversion of urea into ammonia and CO_2_ [97]. However, *N. europaea* has not been reported to grow on urea and urea carboxylase can also serve as a component of a pyrimidine nucleic acid precursor degradation pathway [97]. We regard this as a more likely function in Nir1-infected Nm50^T^ considering phage-induced intracellular nitrogen salvage and redistribution to meet the high nitrogen demand of viral progeny [98]. Alternatively, the urea carboxylase plus adjacent genes might have a potential role in the guanidine metabolism as proposed recently [99].

## Conclusions

Our study provides the first detailed description of phage-induced host modulation in a chemolithoautotrophic ammonia-oxidizing bacterium. We show that virus-induced intracytoplasmic membrane disintegration simultaneously affects lipid homeostasis, energy conservation, and autotrophic carbon metabolism prior to cell lysis. To counterbalance the demand for reducing equivalents and ATP, upregulation of individual iron-dependent respiratory chain components was indicated to support the enzymatic machinery in the remaining membrane of bloated cells. In parallel, uptake systems for nucleic acids, amino acids, and small organic compounds such as pyruvate likely supported metabolic rewiring of the host to support production of viral progeny. In summary, our results are important in three ways. First, since Nir1 is openly available through DSMZ and infects *N. europaea* as one of the best-studied AOB, the Nir1-Nm50^T^ phage-host system may establish as an important model to understand the biology of AOB-infecting phages. Second, our results provide an important basis and first step to assess the impact phages can have on the performance of WWTPs, especially in the background of highly efficient lysis, varying host ranges, and lipid release. Third, since AOB also play an important role in agricultural systems, leading to fertilizer loss [2] and emissions of the greenhouse gas N_2_O [100, 101], the general knowledge on AOB-infecting phages gained in our study will be important for assessing the potential application of phage cocktails for environmental nitrification control. Future research should resolve the molecular mechanisms governing phage–AOB interactions and quantify the environmental consequences of AOB-infecting viruses across engineered and natural ecosystems.

## Supplementary Material

Supplementary_material_ycag170

## Data Availability

The sequenced genome of *Nitrosomonas europaea* Nm50^T^ (=DSM 28437) has been deposited at DDBJ/ENA/GenBank under the accession PRJNA1375795. The sequenced genome of vB_NeuP-Nir1 has been deposited at DDBJ/ENA/GenBank under the accession PX682272. Transcriptomic sequencing data has been deposited at NCBI SRA under the accession PRJNA1380931. Metabolomic data have been deposited at Fairdomhub (doi:10.15490/FAIRDOMHUB.1.ASSAY.2765.1).
