# Supplementary material for: Viral infection collapses intracytoplasmic membrane integrity and autotrophic metabolism in ammonia-oxidizing Nitrosomonas europaea
Source: ISME Commun. 2026 Jun 18;6(1):ycag170. doi: 10.1093/ismeco/ycag170 (PMC13356812; doi:10.1093/ismeco/ycag170)
Supplement: Supplementary_material_ycag170 [file supplementary_material_ycag170.zip › Papendorf_et_al_supplements_ycag170.pdf]

Supplementary material for

Viral infection collapses intracytoplasmic membrane integrity and autotrophic metabolism in ammonia-oxidizing *Nitrosomonas europaea*

János Papendorf[1], David K. Ngugi[2], Petra Büsing[1], Nicole Reimann[1], Johannes Wittmann[1], Stephanie Peter[1], Richard L. Hahnke[1], Sarah Kirstein[1], Boyke Bunk[1], Manfred Rohde[3], Mathias Müsken[3], Meina Neumann-Schaal[1,4], Michael Pester[1,5*]

[1]Leibniz Institute DSMZ-German Collection of Microorganisms and Cell Cultures, Inhoffenstraße 7B, 38124 Braunschweig, Germany

[2]GEOMAR Helmholtz Centre for Ocean Research Kiel, Wischofstraße 1-3, 24148 Kiel, Germany

[3]Central Facility for Microscopy, Helmholtz Centre for Infection Research, Inhoffenstraße 7, 38124 Braunschweig, Germany

[4]Braunschweig Integrated Centre of Systems Biology (BRICS), TU Braunschweig, Rebenring 56, 38106 Braunschweig, Germany

[5]Chair of Microbial Physiology, Technical University of Munich, Emil-Ramann-Str. 4, 85354 Freising, Germany

[*]**Correspondence:** Michael Pester, Technical University of Munich, Emil-Ramann-Str. 4, 85354 Freising, Germany, e-mail: michael.pester@tum.de, phone: +49 (0)8161 71-5445

Supplementary Material and Methods

Composition of DSMZ medium 1583

The main solution of DSMZ medium 1583 [https://mediadive.dsmz.de, 1] is composed of 535 mg L^{-1} NH_4Cl, 54 mg L^{-1} KH_2PO_4, 74 mg L^{-1} KCl, 49 mg L^{-1} $MgSO_4{\times}7H_2O$, 147 mg L^{-1} $CaCl_2{\times}2H_2O$ and 584 mg L^{-1} NaCl in distilled deionized water. The medium was autoclaved and subsequently amended with 2 mL L^{-1} autoclaved and filtered (0.2 µm pore size, polyethersulfone, Sarstedt AG & Co. KG, Nümbrecht, Germany) cresol red solution (0.5 g L^{-1} in distilled deionized water) and 1 mL L^{-1} autoclaved trace element solution. The latter comprises 975 mL distilled deionized water amended with 25 mL 1M HCl, 45 mg L^{-1} $MnSO_4{\times}4H_2O$, 49 mg L^{-1} H_3BO_3, 43 mg L^{-1} $ZnSO_4{\times}7H_2O$, 37 mg L^{-1} $(NH_4)_6Mo_7O_{24}{\times}4H_2O$, 973 mg L^{-1} $FeSO_4{\times}7H_2O$ and 25 mg L^{-1} $CuSO_4{\times}5H_2O$. The pH of DSMZ medium 1583 was initially adjusted by slow addition of autoclaved 10% w/v $NaHCO_3$ until the color of the pH indicator changed from yellow to pink at pH ~7.8. pH had to be regularly re-adjusted by addition of autoclaved 10% w/v $NaHCO_3$ solution during bacterial growth.

Staining and counting of virions

Staining of virions was performed according to a technique described by Kim et al [2] with minor modifications, as described in the following. Stainless steel Luer lock syringe filter holders were equipped with a 0.45 µm pore size cellulose nitrate support filter (Sartorius, Göttingen, Germany) and a 0.02 µm pore size aluminum oxide (Whatman™ Anodisc™, Global Life Sciences Solutions Operations UK Ltd., Little Chalfont, UK) filter to capture virions. Viral lysate in the host medium (DSMZ medium 1583) was pre-filtered through 0.2 µm pore size polyethersulfone filters (Sarstedt AG & Co. KG) to remove host debris. Thereafter, 1 mL of pre-filtered viral lysate was further filtered through the 0.02 µm aluminum oxide filter in the assembled stainless-steel filter capsule (see above) using a 2.5 mL syringe. As a final step, 1 mL of sterile pre-filtered DSMZ medium 1583 (0.2 µm pore size, polyetherfulfone, Sarstedt AG & Co. KG) was used to flush the syringe and the 0.02 µm aluminum oxide filter. The aluminum oxide filters were then immersed in 0.1% w/v low melting Agarose (TopVision, Thermo Fisher Scientific Baltics UAB, Vilnius, Lithuania) to minimize dislodging of virions from the membrane during staining and

observation. Thereafter, filters were transferred to a microscope slide. Staining of virions was performed by pipetting 100 µL of 1:10,000 diluted SYBR Gold (Invitrogen, Life Technologies Corporation, Eugene, OR, USA in TE Buffer, Thermo Fisher Scientific, Ward Hill, MA, USA) on the filter followed by incubation for 10 min in the dark. Afterwards, the stain was carefully pipetted off. To reduce bleaching of stained virions during observation, the filter was covered using 20 µL of a 4:1 mixture of Citifluor AF1 (Electron Microscopy Sciences, Hatfield, USA) and VECTASHIELD (Vector Laboratories, Burlingame, USA). Investigation was done immediately after staining using a BZ-X810 Microscope (KEYENCE DEUTSCHLAND GmbH, Neu-Isenburg, Germany) equipped with a 470/40 nm excitation and 525/50 nm emission filter set. Pictures of the stained virions were captured using the supplied software package BZ-X Analyzer. Calculations of virion concentrations were performed by adding a counting grid of 10×10 µm or 20×20 µm and extrapolating the results of the filtered volume.

Electron Microscopy

Bacteria were fixed stepwise using a concentrated bacterial suspension in growth medium: First, 25% glutaraldehyde was added (final concentration 2%) and incubated for 30 min, followed by addition of 25% paraformaldehyde (final concentration 5%). SEM and TEM sample preparation for ultrathin sections was done as previously described [3, 4], using an automated critical point dryer CPD300 (Leica Microsystems, Wetzlar, Germany). For SEM preparation, in addition to acetone dehydration, ethanol dehydration was performed without a prior washing step. The sample was fixed on a cover slip overnight due to a low bacterial concentration. Visualization of phages and flagella was done as described in [5], using 0.5% uranyl acetate for negative staining of bacteria and 2% for phages and flagella. Flagella were separated by strong vortexing to disconnect them from bacteria, followed by a two-step centrifugation procedure: first to separate bacteria and second to concentrate flagella (16,060×*g*, 30 min at RT).

Genome sequencing of phage vB_NeuP-Nir1

Short-read Illumina and long-read ONT sequencing were done in parallel. For Illumina sequencing, bacterial debris was removed by centrifugation for 15 min at 14,334×*g* and 15°C. Concentration of phage lysate (3.5 L) was achieved by repeated centrifugation in

33 mL aliquots for 2 h at 36,358×*g* and 4°C. Most of the supernatant was removed by pipetting and pellets were resuspended in residual supernatant at 120 rpm on an orbital shaker at room temperature (RT) for 1 h. Concentrated viral lysates were then pooled, resulting in a final volume of 9 mL. For ONT sequencing, bacterial debris was removed using a 0.2 µm pore size filter (polyethersulfone, Sarstedt). Concentration of phage lysate was achieved by centrifuging 2×20 mL phage lysate in Macrosep 30 kDa centrifugal devices (Pall Corporation, Port Washington, NY, USA) for 30 min at 1,000×*g*. For both, Illumina and ONT sequencing, phage DNA was isolated from concentrated lysates using the Phage DNA Isolation Kit (Norgen Biotek Corp, Thorold, Canada) according to the manufacturer's protocol, including the optional Proteinase K and DNase treatment steps. ONT sequencing was performed on a MinION device using the ligation sequencing kit SQK-LSK114 and the Flongle Flow Cell R10.4.1 (Oxford Nanopore Technologies, Oxford, United Kingdom). Libraries for Illumina sequencing were prepared as previously described [6]. Illumina sequencing was performed on the MiSeq System (Illumina, San Diego, CA, USA) with 2×300 bp read length.

Genome sequencing of *N. europaea* DSM 28437 (=Nm50^T)

DNA of strain Nm50^T was isolated from bacterial biomass collected by centrifugation (9,000×*g*, 30 min, 4°C) using the MasterPure™ Complete DNA & RNA Purification Kit (Lucigen Corp., Middleton, USA). DNA extraction was performed following the manufacturer's instructions for cell samples but using double the volumes mentioned in the protocol. In brief, 2 µL Proteinase K solution were diluted in 600 µL of Tissue and Cell Lysis Solution and added to the bacterial pellet. The sample was mixed thoroughly on a vortex mixer and incubated at 65 C° for 15 min. During incubation, the sample was vortexed every 5 min. This was followed by cooling to 37C° for 5 min, adding 2 µL of 5 mg mL^{-1} RNase A, thorough mixing and incubation at 37C° for 30 min. Thereafter, the sample was placed on ice for 5 min. Afterwards, 300 µL of MPC Protein Precipitation Reagent were added to 600 µL of lysed sample and mixed thoroughly using a vortex mixer. The debris was pelleted by centrifugation at 4 C° for 10 minutes at 10,000×*g*. The supernatant was transferred to a clean 2 mL microcentrifuge tube, discarding the pellet. The supernatant was amended with 1 mL of isopropanol, followed by mixing by repeatedly inverting the microcentrifuge tube. The DNA was then pelleted by centrifugation at 4 C°

for 20 min at 20,817×*g*. The isopropanol was carefully poured off without dislodging the DNA pellet which was then rinsed with freshly prepared 70% ethanol. The residual ethanol was removed with a pipette and the pellet was air-dried. Finally, the DNA was resuspended in 50 µL TE buffer.

SMRTbell® template library was prepared according to the instructions from Pacific Biosciences (Menlo Park, USA), following the Procedure & Checklist – Preparing whole genome and metagenome libraries using SMRTbell® prep kit 3.0. Briefly, for preparation of 10 kb libraries 2 µg genomic DNA were sheared using the Megaruptor® 3 (Diagenode, Denville, NJ, USA) according to the manufacturer´s instructions. DNA was end-repaired and ligated to barcoded adapters applying components from the SMRTbell® prep kit 3.0 (Pacific Biosciences). Reactions were carried out according to the manufacturer´s instructions. Samples were pooled equimolar. Conditions for annealing of sequencing primers and binding of polymerase to purified SMRTbell® template were assessed according to the Sample Setup in SMRT®link (Pacific Biosciences). Libraries were sequenced on the Revio System (Pacific Biosciences) taking one 30h movie per SMRT cell. Long read genome assembly was performed using the "Microbial Genome Analysis" protocol included in SMRTlink version 13.1 using default parameters. Herewith, one circular bacterial chromosome was obtained. Annotation of the genome was performed using the NCBI prokaryotic genome annotation pipeline [7].

Diagnostic PCR assay for the gene encoding the major head protein of Nir1

At the WWTP Steinhof, secondary clarifiers are fed by three effluents from corresponding aeration basins. Each effluent was sampled once per month for twelve consecutive months (February 2024 – January 2025). Samples were collected using a custom rod sampler, which was flushed three times in the wastewater stream before sampling. Using serological pipettes, 25 mL of wastewater were transferred to 50 mL conical polypropylene centrifuge tubes (TPP Techno Plastic Products AG, Trasadingen, Switzerland), transported to the laboratory and frozen at −20°C until analysis. After thawing, samples were vortexed for 30 sec to resuspend the flocculated activated sludge. DNA extraction was performed on a 200 µL subset of activated sludge from each sample using the AllPrep PowerViral DNA/RNA Kit (Qiagen, Hilden, Germany) according to the manufacturer's protocol, including beat beating to ensure complete lysis.

Primer-BLAST [8] was used to design the PCR primer pair cap3f (5’-TGCTACGGAGGCTAAAGCTCG-3’) and cap3r (5’-CTACTGCATCACCACGCAGC-3’), targeting a 170-bp long region of the gene encoding the major head protein of Nir1. A specificity check via Primer-BLAST against the NCBI nucleotide collection (nt) database identified only the highly similar gene of phage ΦNF-1 (OL634959.1) as additional potential target, with a single nucleotide mismatch for one of the two primers. PCR was performed with 60 ng template, 0.4 µL of each primer (10 µM), 10 µL 2× DreamTaq Green PCR Master Mix (Thermo Fisher Scientific Baltics UAB) and a total volume of 20 µL on a Biometra TOne (Analytik Jena GmbH+Co. KG, Jena, Germany) thermocycler. Genomic DNA (0.121 ng) of Nir1 served as positive control. Molecular grade, nuclease-free water (Ambion™, Life Technologies Corporation, Austin, TX, USA) served as negative control. PCR conditions were: 95°C initial denaturation, 35 cycles of 95°C, 60°C, 72°C each for 30 sec, and 5 min of final elongation at 72°C.

Host range experiments

All bacterial strains were grown in DSMZ medium 1583 at 28°C in the dark without agitation. Actively growing cultures (n=3) of the AOBs *Nitrosomonas eutropha* C-91^T (=DSM 101675, NCBI acc. CP000450, average nucleotide identity (ANI) of 79.26% to Nm50^T), *Nitrosomonas communis* Nm2^T (=DSM 28436, NCBI acc. CP011451, ANI of 69.92% to Nm50^T), *Nitrosomonas nitrosa* Nm90^T (=DSM 28438, NCBI acc. QAOO01000001, ANI of 69.67% to Nm50^T) and *Nitrosospira multiformis* C-71^T (=DSM 101674, NCBI acc. CP000103, ANI of 68.83% to Nm50^T) were amended with 0.2-µm-filtered viral lysate of Nir1. Strain Nm50^T was used as positive control to confirm phage infectivity. After three and seven days, all cultures were examined for color change of the pH indicator as an indicator for ammonia-oxidizing activity and, in addition, by phase contrast microscopy for bloated and lysed cells. ANI values were calculated based on the OrthoANIu approach using CJ Bioscience’s web-based ANI Calculator [9] based on the OrthoANI algorithm [10].

Analysis of the latent period of Nir1

Duration of the latent period of Nir1 and onset of lysis of infected *N. europaea* $Nm50^T$ cells were analyzed using the following procedure. An $Nm50^T$ culture was grown statically in a 1 L Erlenmeyer flask at 28°C in the dark. Directly prior to the experiment, 30 mL aliquots were transferred to eight 50 mL Erlenmeyer flasks. Four replicates served as uninfected controls. The remaining four replicates were amended with 2.0 mL phage lysate of Nir1 (MOI = 4.1). Sampling to determine bacterial concentrations in $Nm50^T$ cultures was performed directly at the start of each treatment and after 300 min. Infected cultures were additionally sampled 120 min, 180 min and 240 min post infection. Samples to analyze extracellular phage concentrations of Nir1 were taken from infected cultures directly after addition of lysate and every 30 min until 300 min post infection. Control samples were collected from uninfected cultures directly at the start of the experiment and treated equally to samples from infected cultures. All samples were analyzed using a CytoFLEX S (Beckman Coulter, Brea, CA, USA) benchtop flow cytometer with 4 µm CountBright Plus Absolute Counting Beads (Invitrogen) serving as internal standard. Bacterial concentrations were determined as stated in the main manuscript. Phage concentrations were determined as follows. Samples were directly filtered (0.2 µm pore size, polyethersulfone, Sarstedt AG & Co. KG) after sampling and stored at 5°C until analysis. Samples were then diluted 1:100 using pre-filtered DSMZ medium 1583 (0.2 µm pore-size, polyethersulfone, Sarstedt AG & Co. KG). Thereafter, 999 µL were stained for 10 min in the dark using 1 µL of a 1:10 dilution of SYBR Gold (Invitrogen, diluted in pre-filtered DSMZ medium). To determine phage concentrations, a threshold of 2,000 in the SYTO-9-H channel and adjusted gain settings (forward scatter FSC=120, violet side scatter VSSC=300, SYTO-9=1750) were used. Events representing virions and counting beads were captured in gates created in SYTO-9 vs VSSC and FSC vs. SYTO-9 plots, respectively. Phage counts in infected cultures were corrected for background noise at the same gate settings in control cultures (n=3) to account for background noise. Flow cytometry-based phage concentrations were benchmarked against microscopic counts of SYBR Gold-stained virions captured on aluminum oxide filters (Whatman™ Anodisc™, Global Life Sciences Solutions Operations UK Ltd.). This was done with samples of infected cultures taken directly after the start of infection ($2.33\times10^7 \pm 7.82\times10^6$, n=4, mean ± standard deviation), 120 min post infection ($1.17\times10^7 \pm 1.59\times10^6$, n=3, mean ± standard

deviation) and 300 min post infection ($5.18 \times 10^8 \pm 1.21 \times 10^8$, n=4, mean ± standard deviation), which confirmed that flow cytometer counts were in the correct order of magnitude.

Supplementary Figures

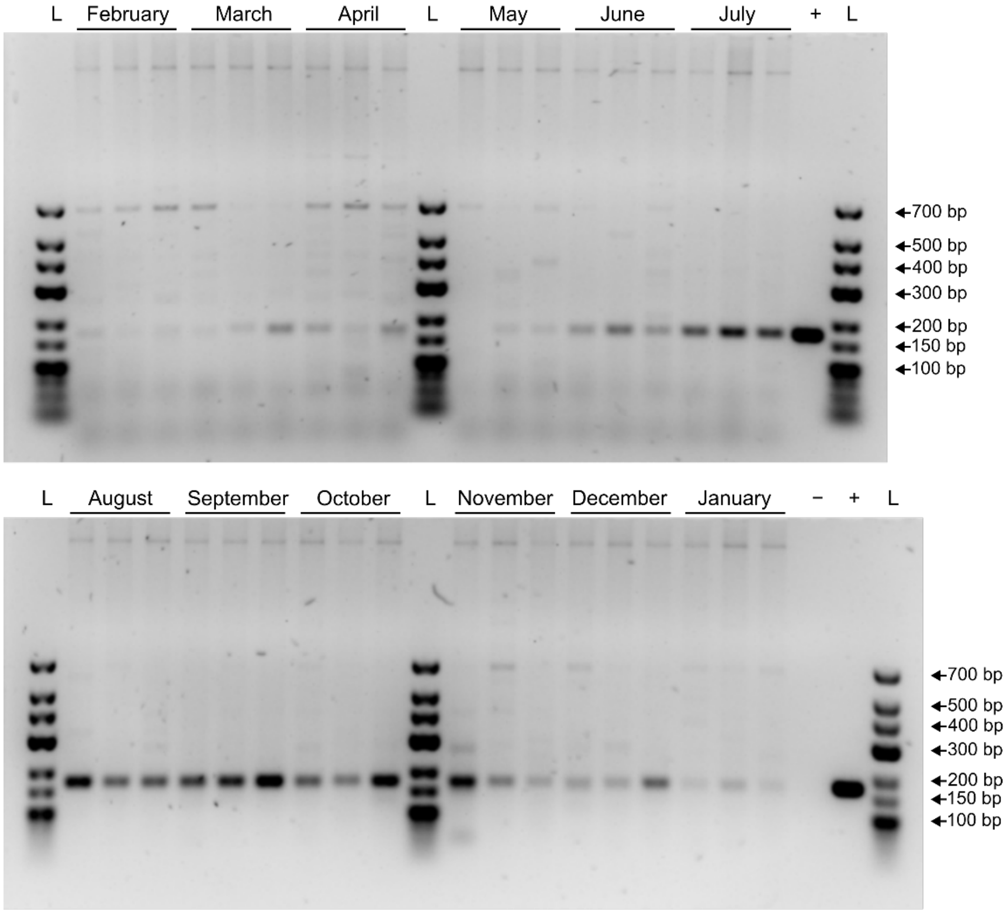

Supplementary Figure 1. Products of a PCR-amplified 170 bp long region of the major head protein of phage vB_NeuP-Nir1, separated by agarose gel electrophoresis using 2% w/v Wide Range agarose (SERVA Electrophoresis GmbH, Heidelberg, Germany) in 1× TAE buffer (Carl Roth GmbH + Co. KG, Karlsruhe, Germany). Electrophoresis was run for 75 min at 130 V. Negative control (-) = nuclease-free water (Thermo Fisher Scientific Baltics UAB, Vilnius, Lithuania), positive control (+) = 0.121 ng genomic DNA of phage vB_NeuP-Nir1, Ladder (L) = GeneRuler Low Range DNA Ladder (Thermo Fisher Scientific Baltics UAB).

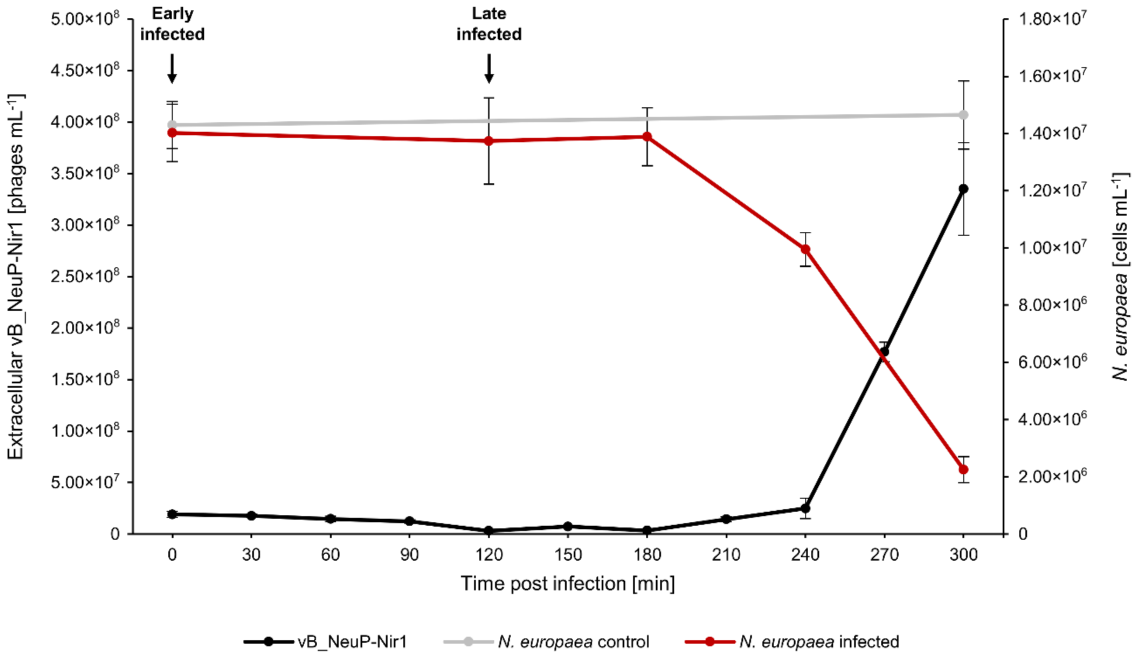

Supplementary Figure 2. Analysis of the latent period of phage vB_NeuP-Nir1 until onset of lysis of infected *Nitrosomonas europaea* Nm50^T cells (MOI = 4.1). Bacterial concentrations of *N. europaea* in infected and control cultures and extracellular phage vB_NeuP-Nir1 concentrations in infected cultures were determined using flow cytometry. Data is shown as the mean (n=4), error bars represent standard deviation. Time points, chosen for sampling during the respective experiments analyzing transcriptomic or metabolomic responses of Nir1-infected Nm50^T cultures, are marked with black arrows.

Supplementary Tables

Supplementary Table 1. Differentially expressed genes of Nir1-infected *N. europaea* Nm50^T compared to non-infected controls during the late infection process (sampled 120 min post infection). Genes are marked according to their assigned functional category. **(→Separate Excel file)**

Supplementary Table 2. Comparison of amino acid frequencies in total CDS of the host bacterium *N. europaea* Nm50^T, total CDS of the infecting bacteriophage vB_NeuP-Nir1 (Nir1) and major head protein CDS of Nir1.

Amino acid	Amino acid single letter	Frequency in Nm50^T CDS	Frequency in Nir1 CDS	Frequency in Nir1 major head protein CDS
Alanine	A	9.20%	7.90%	10.80%
Cysteine	C	1.00%	0.90%	0.30%
Aspartate	D	5.30%	5.90%	7.00%
Glutamate	E	6.00%	6.10%	4.40%
Phenylalanine	F	3.90%	3.20%	4.40%
Glycine	G	7.20%	7.60%	7.30%
Histidine	H	2.50%	1.90%	2.60%
Isoleucine	I	6.30%	5.50%	4.40%
Lysine	K	4.10%	5.60%	4.70%
Leucine	L	10.60%	8.50%	7.90%
Methionine	M	2.40%	3.10%	2.60%
Asparagine	N	3.60%	4.50%	4.70%
Proline	P	4.60%	4.00%	3.20%
Glutamine	Q	4.30%	3.70%	3.80%
Arginine	R	6.40%	5.60%	5.60%
Serine	S	6.00%	7.40%	6.10%
Threonine	T	5.50%	5.70%	8.50%
Valine	V	6.70%	7.10%	6.70%
Tryptophan	W	1.30%	1.50%	2.00%
Tyrosine	Y	2.90%	3.90%	2.60%
STOP	*	0.30%	0.50%	0.30%

Supplementary References

1. Koblitz J, Halama P, Spring S, Thiel V, Baschien C, Hahnke RL *et al.* MediaDive: the expert-curated cultivation media database. *Nucleic Acids Res* 2023;**51**:D1531-D38. https://doi.org/10.1093/nar/gkac803
2. Kim JG, Kim SJ, Cvirkaite-Krupovic V, Yu WJ, Gwak JH, Lopez-Perez M *et al.* Spindle-shaped viruses infect marine ammonia-oxidizing thaumarchaea. *Proc Natl Acad Sci U S A* 2019;**116**:15645-50. https://doi.org/10.1073/pnas.1905682116
3. Vieira S, Pascual J, Boedeker C, Geppert A, Riedel T, Rohde M *et al. Terricaulis silvestris* gen. nov., sp. nov., a novel prosthecate, budding member of the family *Caulobacteraceae* isolated from forest soil. *Int J Syst Evol Microbiol* 2020;**70**:4966-77. https://doi.org/10.1099/ijsem.0.004367
4. Vieira S, Huber KJ, Geppert A, Wolf J, Neumann-Schaal M, Luckner M *et al. Capillimicrobium parvum* gen. nov., sp. nov., a novel representative of *Capillimicrobiaceae* fam. nov. within the order *Solirubrobacterales*, isolated from a grassland soil. *Int J Syst Evol Microbiol* 2022;**72** https://doi.org/10.1099/ijsem.0.005508
5. Wittmann J, Dreiseikelmann B, Rohde C, Rohde M, Sikorski J. Isolation and characterization of numerous novel phages targeting diverse strains of the ubiquitous and opportunistic pathogen *Achromobacter xylosoxidans*. *PLoS One* 2014;**9**:e86935. https://doi.org/10.1371/journal.pone.0086935
6. Korf IHE, Meier-Kolthoff JP, Adriaenssens EM, Kropinski AM, Nimtz M, Rohde M *et al.* Still Something to Discover: Novel Insights into *Escherichia coli* Phage Diversity and Taxonomy. *Viruses* 2019;**11** https://doi.org/10.3390/v11050454
7. Tatusova T, DiCuccio M, Badretdin A, Chetvernin V, Nawrocki EP, Zaslavsky L *et al.* NCBI prokaryotic genome annotation pipeline. *Nucleic Acids Res* 2016;**44**:6614-24. https://doi.org/10.1093/nar/gkw569
8. Ye J, Coulouris G, Zaretskaya I, Cutcutache I, Rozen S, Madden TL. Primer-BLAST: A tool to design target-specific primers for polymerase chain reaction. *BMC Bioinformatics* 2012;**13**:134. https://doi.org/10.1186/1471-2105-13-134
9. Yoon SH, Ha SM, Lim J, Kwon S, Chun J. A large-scale evaluation of algorithms to calculate average nucleotide identity. *Antonie Van Leeuwenhoek* 2017;**110**:1281-86. https://doi.org/10.1007/s10482-017-0844-4
10. Lee I, Ouk Kim Y, Park S-C, Chun J. OrthoANI: An improved algorithm and software for calculating average nucleotide identity. *Int J Syst Evol Microbiol* 2016;**66**:1100-03. https://doi.org/https://doi.org/10.1099/ijsem.0.000760
